# Successful Open Surgical Repair of Traumatic Stanford Type B Thoracic Aortic Dissection Following Motorcycle Trauma: A Case Report

**DOI:** 10.7759/cureus.105408

**Published:** 2026-03-17

**Authors:** Nabil Y Al_Madhwahi, Ali M Fadhel, Yahya A Al-Modwahi, Al-Ezy M Hamoud, Emad A Halboob

**Affiliations:** 1 Department of Vascular Surgery, Faculty of Medicine and Health Sciences, Sana’a University, Sana'a, YEM; 2 Department of Cardiac Surgery, Al-Thawra Modern General Hospital, Sana’a, YEM; 3 Department of Vascular Surgery, Al-Thawra Modern General Hospital, Sana'a, YEM

**Keywords:** aortic dissection, dacron graft, emergency surgery, stanford type b, thoracic aorta, thoracic aortic dissection, traumatic aortic injury, vascular surgery

## Abstract

Traumatic thoracic aortic injury represents one of the most life-threatening complications of blunt chest trauma and is associated with substantial morbidity and mortality if not promptly recognized and treated. Rapid deceleration mechanisms may produce intimal disruption of the aortic wall, resulting in dissection, false lumen formation, and potential aortic rupture. We report the case of an 18-year-old previously healthy male patient who presented with chest pain and hemothorax three days after a motorcycle accident. Computed tomography angiography (CTA) demonstrated a Stanford type B traumatic aortic dissection, characterized by an intimal flap originating approximately 3 cm distal to the left subclavian artery and extending along the proximal descending thoracic aorta. The maximal aortic diameter measured approximately 2.6 cm, which was within the expected range for the patient's age. The patient was successfully treated with emergency open surgical repair through a left posterolateral thoracotomy with interposition of a Dacron graft. Postoperative recovery was uneventful, and follow-up imaging confirmed graft patency with restoration of normal aortic flow. The patient was discharged on postoperative day 14 in a stable condition. This case highlights the importance of early diagnosis and timely surgical intervention in traumatic thoracic aortic dissection. In resource-limited settings where endovascular therapy may not be available, conventional open surgical repair remains a reliable and life-saving therapeutic option.

## Introduction

Blunt thoracic aortic injury represents one of the most lethal complications of blunt trauma and remains a major cause of mortality in severely injured patients. It is considered the second leading cause of death in blunt trauma after traumatic brain injury [1]. The reported incidence of blunt aortic injury among trauma patients ranges between 0.5% and 2%, with motor vehicle collisions accounting for the majority of cases due to high‑energy deceleration mechanisms [2-4]. Historically, a substantial proportion of patients with traumatic aortic injury die at the scene of the accident, and among those who survive long enough to reach the hospital, mortality remains significant without timely diagnosis and intervention [1,3].

Stanford type B aortic dissection involves the descending thoracic aorta distal to the origin of the left subclavian artery and represents an important subset of traumatic thoracic aortic injuries [5]. The pathophysiology of blunt traumatic aortic injury is primarily related to rapid deceleration forces that generate significant shear stress on the aortic wall, particularly at anatomical fixation points such as the aortic isthmus near the ligamentum arteriosum [1,3]. These forces may result in intimal tears that allow blood to enter the medial layer, producing a dissecting flap and formation of a false lumen. Progressive propagation of the dissection may subsequently lead to an aortic rupture or a compromised perfusion of branch vessels with resulting organ ischemia [6].

Advances in imaging modalities, particularly computed tomography angiography (CTA), have significantly improved the early detection of traumatic aortic injuries and have become the diagnostic modality of choice in hemodynamically stable trauma patients [7]. Management strategies have also evolved considerably over the past two decades. Thoracic endovascular aortic repair (TEVAR) has emerged as the preferred treatment modality in many centers because of the lower perioperative morbidity and reduced operative time compared with conventional open repair [2,8]. Nevertheless, open surgical repair with prosthetic graft replacement remains an important therapeutic option, particularly in resource‑limited settings where endovascular technology may not be readily available [1,3,8]. 

Herein, we report a case of an adult male patient with a traumatic Stanford type B thoracic aortic dissection, which developed following a motorcycle accident and was successfully managed by emergency open surgical repair with a synthetic graft placement. This case is particularly noteworthy because, in addition to the delayed presentation, it demonstrates successful open surgical management of a traumatic Stanford type B thoracic aortic dissection in a young patient within a resource-limited healthcare setting where TEVAR was not available.

## Case presentation

An 18-year-old previously healthy male farmer from Sa'adah, Yemen, presented to the emergency department three days after a motorcycle accident. The patient was thrown from his motorcycle following a collision with two cars and sustained multiple traumatic injuries. He initially received treatment at a rural hospital before being referred to our center for further evaluation. His medical history was unremarkable, with no history of hypertension, diabetes mellitus, cardiovascular disease, or prior surgical procedures. There was no family history of cardiovascular disease or connective tissue disorders.

On admission, the patient complained of sharp left-sided chest pain aggravated by deep inspiration and associated with mild shortness of breath. There was no history of loss of consciousness, vomiting, or seizures following the accident.

On physical examination, the patient was conscious and oriented to time, place, and person. He appeared pale but had no jaundice or cyanosis. Mild respiratory distress was noted, although the patient remained cooperative and responsive. Vital signs were stable with a blood pressure of 110/70 mmHg, heart rate of 92 beats per minute, respiratory rate of 20 breaths per minute, and oxygen saturation of 97% with supplemental oxygen. 

Chest examination revealed asymmetric chest expansion with reduced movement on the left side, diminished air entry over the left lung field, and dullness to percussion over the left hemithorax. No rib tenderness was detected. Cardiovascular examination revealed normal first and second heart sounds (S1 and S2) without murmurs, rubs, or gallops. No peripheral edema was present. Transthoracic echocardiography was performed and demonstrated normal cardiac function with no evidence of valvular abnormalities, pericardial effusion, or cardiac injury.

**Figure 1:**
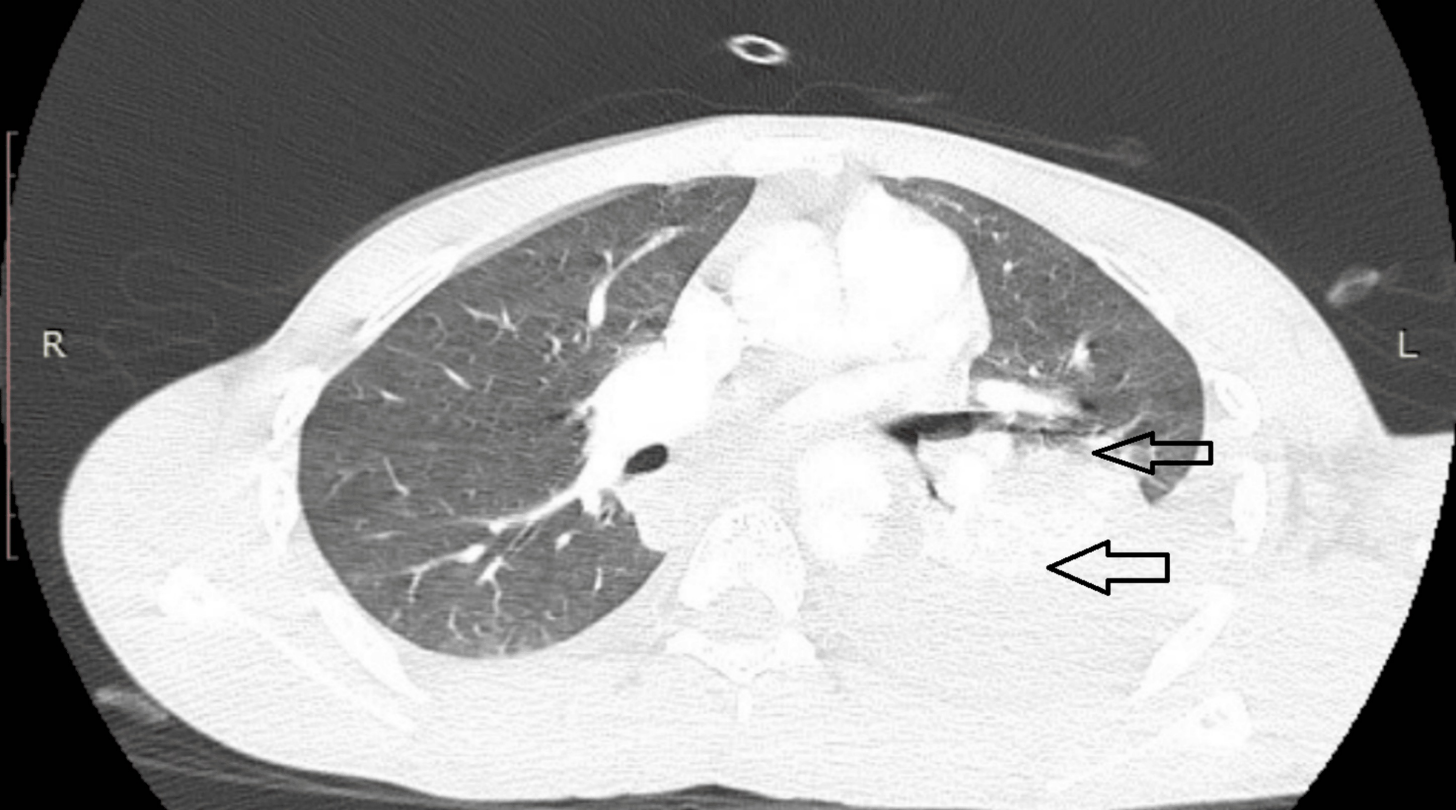
Preoperative computed tomography (CT) imaging Axial lung-window CT images demonstrating left-sided hemothorax with partial collapse of the left lung, consistent with traumatic injury following blunt chest trauma (arrows). The right lung appears relatively well aerated.

A comprehensive trauma evaluation revealed no evidence of associated injuries such as head trauma, abdominal injury, or pelvic fractures. Preoperative CT imaging demonstrated a left-sided hemothorax with a partial collapse of the left lung (Figure 1). 

**Figure 2:**
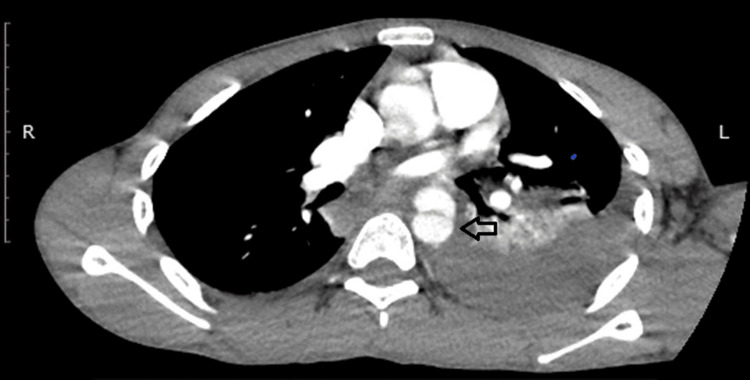
Preoperative computed tomography angiography (CTA) imaging Axial contrast-enhanced CTA images showing an intimal flap within the proximal descending thoracic aorta, creating true and false lumens consistent with a traumatic Stanford type B aortic dissection (arrow).

**Figure 3:**
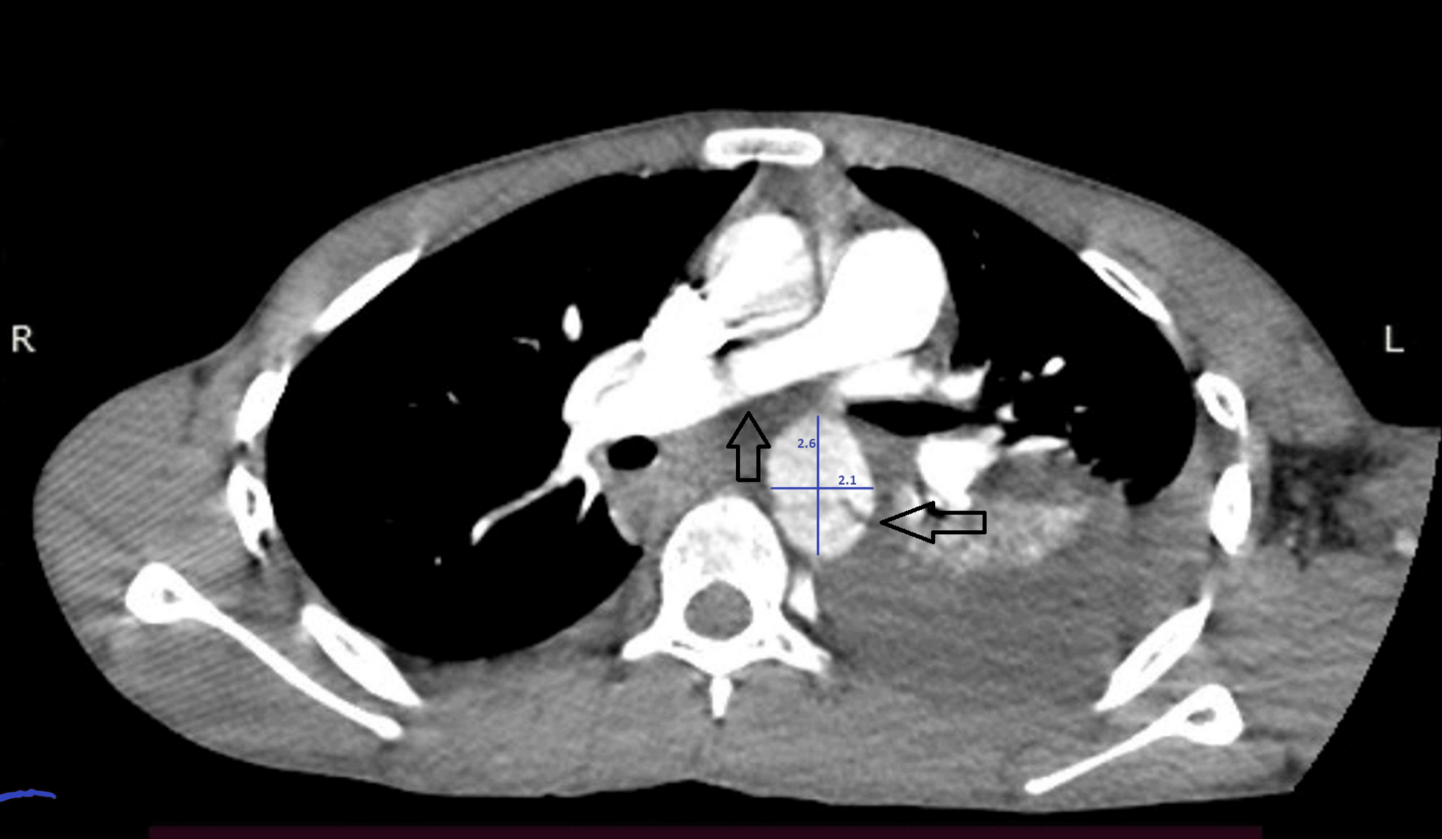
Preoperative computed tomography angiography (CTA) imaging Axial contrast-enhanced CTA images showing an intimal flap within the proximal descending thoracic aorta distal to the origin of the left subclavian artery, creating true and false lumens consistent with a traumatic Stanford type B aortic dissection (arrows). An associated left pleural hemothorax is also visualized.

Contrast-enhanced CTA further revealed an intimal flap within the proximal descending thoracic aorta distal to the origin of the left subclavian artery, forming true and false lumens consistent with a traumatic Stanford type B aortic dissection with a 2.6 cm maximum diameter of the aorta (Figures 2, 3).

The lesion originated approximately 3 cm distal to the origin of the left subclavian artery and extended along the descending thoracic aorta. No active contrast extravasation or mediastinal hematoma, suggestive of free rupture, was identified.

**Figure 4:**
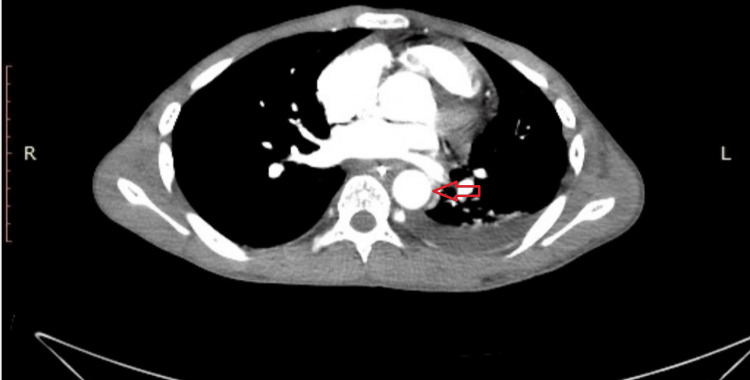
Postoperative computed tomography angiography (CTA) image Axial CTA image of the chest demonstrates the successful exclusion of the traumatic aortic dissection. The image shows a patent graft in the descending thoracic aorta with no evidence of a graft leak, pseudoaneurysm, or other graft-related complications. The aortic contour is regular, and there is complete resolution of the false lumen (arrow). A residual, non-expanding left-sided pleural effusion is also noted, which is a common finding following thoracotomy.

Given the diagnosis of traumatic Stanford type B aortic dissection involving the proximal descending thoracic aorta, emergency surgical intervention was indicated due to the risk of progression, rupture, and other life-threatening complications. Open surgical repair was therefore selected as the primary treatment strategy to achieve definitive exclusion of the intimal tear and restoration of normal aortic integrity. Early postoperative CTA demonstrated satisfactory graft position without evidence of thoracic aortic re-dilatation (Figure 4).

Long-term surveillance with periodic CTA was planned.

Treatment

The patient was prepared for emergency surgical intervention after consultation with the anesthesia and cardiovascular surgery teams. Invasive hemodynamic monitoring was established, including arterial and central venous lines, and a urinary catheter was inserted for urine output monitoring. The patient underwent general anesthesia with endotracheal intubation and mechanical ventilation.

A left posterolateral thoracotomy was performed through the fifth intercostal space to expose the descending thoracic aorta. After entering the pleural cavity, the left lung was gently retracted to allow adequate visualization of the aorta. Proximal and distal control of the aorta was obtained using vascular clamps placed above and below the dissected segment.

Partial left heart bypass was established to maintain distal perfusion during aortic cross-clamping and to reduce the risk of spinal cord ischemia. The affected aortic segment was opened, revealing the false lumen and the intimal flap. The torn intimal flap was carefully resected to eliminate the dissected segment and restore the integrity of the aortic wall.

A Dacron synthetic vascular graft (JOTEC GmbH, Hechingen, Germany) of appropriate size was selected and interposed to replace the diseased segment of the descending thoracic aorta. The graft was positioned to span the entire dissected area, beginning approximately 3 cm distal to the origin of the left subclavian artery and extending distally along the descending thoracic aorta. Proximal and distal anastomoses were performed using continuous 5-0 polypropylene sutures (Prolene^TM^ polypropylene sutures, Ethical Inc., Johnson & Somerville, NJ, USA), ensuring secure and hemostatic connections.

After completion of the anastomoses, the vascular clamps were gradually released to restore aortic blood flow. Careful inspection confirmed adequate hemostasis and satisfactory distal perfusion. A chest drain was placed in the pleural cavity, and the thoracotomy incision was closed in layers. The total operation time was approximately three hours, and no intraoperative complications were observed.

Postoperatively, the patient was transferred to the intensive care unit for close monitoring. Hemodynamic stability was maintained with careful blood pressure control to minimize stress on the aortic repair. Pain control, antibiotic prophylaxis, and respiratory physiotherapy were initiated to facilitate recovery and prevent postoperative pulmonary complications.

The patient’s postoperative course was uneventful. Follow-up CTA was obtained on postoperative day 10 which confirmed satisfactory graft placement with restoration of normal aortic flow and no evidence of graft-related complications (Figure 4).

The patient was transferred from the intensive care unit on postoperative day five and discharged from the hospital on postoperative day 14 in stable condition.

Long-term follow-up with periodic CTA was recommended to monitor graft integrity and detect potential late complications such as pseudoaneurysm formation or progressive aortic dilation. The patient was advised to avoid heavy physical activity during the early postoperative period and to maintain regular clinical follow-up.

## Discussion

Traumatic thoracic aortic injury remains one of the most devastating complications of blunt chest trauma and continues to be associated with substantial morbidity and mortality despite significant advances in trauma systems and critical care management [1]. High-energy deceleration mechanisms, such as motor vehicle collisions, generate intense shear forces on the aortic wall. These forces are particularly concentrated at the aortic isthmus near the ligamentum arteriosum, where the relatively fixed descending thoracic aorta meets the mobile aortic arch, making this region the most common site of injury [1,3]. The resulting mechanical stress may lead to intimal disruption, allowing blood to penetrate the medial layer and creating a dissection plane with formation of true and false lumens [6].

The clinical presentation of traumatic aortic injury can be highly variable and frequently nonspecific, particularly in patients with multiple associated injuries [1]. Symptoms may include chest pain, dyspnea, or findings related to concomitant thoracic trauma such as hemothorax or pulmonary contusion [9]. Consequently, a high index of suspicion is required in patients presenting after high-energy blunt trauma. In the present case, the combination of chest pain and hemothorax following a motorcycle collision raised concern for a major thoracic vascular injury and prompted further diagnostic evaluation.

CTA has become the diagnostic modality of choice for suspected traumatic thoracic aortic injury due to its excellent sensitivity, specificity, and rapid availability in modern trauma centers [2,7]. CTA provides detailed visualization of the aortic wall and allows for accurate identification of characteristic findings, such as intimal flaps, pseudoaneurysm formation, intramural hematoma, and extent of aortic involvement [4]. In our patient, CTA clearly demonstrated a Stanford type B traumatic aortic dissection, originating in the proximal descending thoracic aorta distal to the left subclavian artery.

Management strategies for traumatic thoracic aortic injury have evolved considerably over the past two decades. Historically, treatment consisted of strict medical management aimed at reducing aortic wall stress through aggressive blood pressure and heart rate control [10]. Although medical stabilization remains an important component of early management, definitive repair is generally recommended for patients with significant aortic injury due to the risk of progression and delayed rupture [2].

In recent years, TEVAR has emerged as the preferred treatment modality in many institutions because it is associated with lower perioperative morbidity and mortality compared with conventional open repair [3,11]. Multiple studies and systematic reviews have demonstrated improved early outcomes with TEVAR, including reduced operative time, decreased blood loss, and a lower incidence of neurologic complications such as paraplegia [4]. Accordingly, contemporary guidelines from major cardiovascular societies like the American Heart Association and European Society of Cardiology recommend endovascular repair as the first-line treatment for suitable patients with traumatic thoracic aortic injury [12].

Despite these advances, open surgical repair remains an essential therapeutic option in selected clinical scenarios. This is particularly relevant in resource-limited environments where endovascular technology and specialized expertise may not be readily available. Furthermore, open surgical repair may also be preferred in selected younger patients because of the long-term durability of surgical grafts. In addition, certain anatomical considerations may preclude safe endovascular repair. Open surgical repair with prosthetic graft interposition therefore continues to represent a durable and well-established treatment strategy. Several studies have demonstrated favorable long-term outcomes and durable aortic reconstruction following open repair for traumatic thoracic aortic injury [1,11].

In the present case, emergency open surgical repair through a left posterolateral thoracotomy with interposition of a Dacron graft was successfully performed. Intraoperative strategies, including partial left heart bypass and careful control of aortic cross-clamping, were employed to maintain distal perfusion and minimize the risk of spinal cord ischemia. The patient had an uneventful postoperative recovery, and follow-up imaging confirmed satisfactory graft patency with restoration of normal aortic flow.

This case highlights that traumatic thoracic aortic injuries may occasionally remain clinically stable for several days before diagnosis, underscoring the importance of maintaining a high index of suspicion in patients presenting after high-energy blunt trauma. Early diagnosis with CTA and prompt definitive intervention remain critical determinants of survival. Furthermore, our experience demonstrates that, in settings where endovascular resources are limited, conventional open surgical repair remains a safe, effective, and life-saving treatment strategy.

## Conclusions

Traumatic thoracic aortic dissection represents a critical vascular emergency following high-energy blunt chest trauma. Early diagnosis using CTA and prompt definitive intervention are essential to prevent fatal complications. This case demonstrates that emergency open surgical repair with prosthetic graft interposition can achieve excellent outcomes in young patients with traumatic Stanford type B aortic dissection. Importantly, in resource-limited environments where endovascular therapy is not readily available, conventional open surgical repair remains a reliable and life-saving treatment strategy.
